# An in-depth study on survival mechanism of bacterial isolates in disinfectants within the hospital environment

**DOI:** 10.3389/fcimb.2024.1442914

**Published:** 2024-08-15

**Authors:** Pue Rakshit, Aradhana Singh, Ravindra Singh, Tuhina Banerjee

**Affiliations:** ^1^ Department of Microbiology, Institute of Medical Sciences, Banaras Hindu University, Varanasi, Uttar Pradesh, India; ^2^ Trauma Center, Institute of Medical Sciences, Banaras Hindu University, Varanasi, Uttar Pradesh, India

**Keywords:** disinfectant resistance, MBC, WGS, chlorhexidine, benzalkonium chloride, biofilm

## Abstract

**Introduction:**

The emergence of disinfectant resistance has become a severe threat due to reduced effectiveness. This study was undertaken to determine how bacteria adapt to survive exposure to disinfectants in the busiest section of a tertiary care hospital in Varanasi, India.

**Methods:**

Four isolates (two *Klebsiella pneumoniae*, Kp1 and Kp2; two *Pseudomonas aeruginosa*, Pa1 and Pa2) were obtained from chlorhexidine (CHX)–based handwash during microbiological surveillance of “in-use disinfectants” in hospital. Six disinfectants [4% CHX, 2% glutaraldehyde, 7.5% hydrogen peroxide, 1% sodium hypochlorite and 0.1% benzalkonium chloride (BAC), and 70% ethyl alcohol] were tested against these four isolates to determine minimum inhibitory concentration (MIC) and minimum bactericidal concentration (MBC). Antibiotic profile, change in MIC on exposure to disinfectants and biofilm formation in the presence and absence of disinfectants was studied. Whole genome sequencing (WGS) was done to identify the resistance mechanisms.

**Result:**

The isolates showed the highest MBC/MIC ratio (4) against glutaraldehyde. Exposure to supra-inhibitory concentration of BAC for 21 days resulted in doubling of MIC/MBC. The majority (75%) of the isolates were multidrug resistant. All the isolates were strong biofilm producers. The reduction rate of biofilm formation decreased with an increase in the concentration of disinfectants (*p* = 0.05 for BAC). WGS revealed multiple AMR genes including *bla*_DIM-1_, disinfectant-resistant gene and efflux pump genes.

**Conclusion:**

The study emphasized the various adaptation strategies of these isolates for survival in disinfectant environment, thus posing a huge challenge for their control in the hospital environment.

## 1 Introduction

The rapid emergence of antimicrobial resistance (AMR) is a global threat (Prestinaci et al., 2015). Along with it, there has been a parallel increase in disinfectant resistance resulting in their reduced effectiveness (Rozman et al., 2021). The driving force behind the sudden emergence of disinfectant resistance can be largely attributed to the abuse and misuse of disinfectants as well as lack of understanding of their resistance mechanisms (Carlie et al., 2020). The absence of a widely accepted definition for resistance to disinfectants is a critical hurdle, which might have contributed to the limited attention received from practitioners, administrators, and authorities on this challenging issue (Harbarth et al., 2014). Microorganisms, often dwell on hospital surfaces including patient surroundings and in biofilms where disinfectants cannot reach effortlessly (Tezel and Pavlostathis, 2015). The concentrations of disinfectants utilized in practice are much higher than the minimum inhibitory concentration (MIC) values, which often goes unnoticed (van Dijk and Verbrugh, 2022).

During routine microbiological surveillance in a tertiary care hospital, Gram-negative bacteria were found in “in-use” handwash. Consequently, we hypothesized that these isolates were adapted to survival mechanisms to overcome the disinfectant effect. With this background, the following study was undertaken to study in detail the adaptive strategies employed by the bacterial isolates to survive disinfectant exposures in the hospital environment through a series of phenotypic and genotypic techniques.

## 2 Materials and methods

### 2.1 Study site

This descriptive study was conducted in the Department of Microbiology and the associated tertiary care hospital in Varanasi, North India.

### 2.2 Bacterial isolates identification

During the microbiological surveillance of “in-use disinfectants” from major wards and intensive care units of the tertiary care hospital, four bacterial isolates were obtained from chlorhexidine (CHX)–based handwash. These were isolated from aseptically collected disinfectants from the dispensers in more than one of the busiest areas of the hospital with high patient footfall. Disinfectants were inoculated in blood agar and MacConkey agar (HiMedia Laboratories Pvt Ltd, India) and incubated at 37°C. Organisms were identified by standard biochemical tests (Crichton, 1996). The lot number of the dispensers was checked and noted.

### 2.3 In-use testing of disinfectant contamination

To check the “in-use” CHX-based disinfectants, “in-use testing” was done for the entire batch from the store with the same lot number as well as from those collected in the surveillance. One milliliter of CHX-based disinfectant was added to 9 mL of the media along with 1mL of 5% (w/v) autoclaved yeast suspension. A volume of 0.02 mL was placed on each of the two nutrient agar plates. One of the plates was incubated at 37°C for 3 days and the other at room temperature for 7 days. Five or more colonies on each plate indicated contamination (Rao S, 2008).

### 2.4 Determination of MIC against disinfectants

This was done to determine the MIC of the four isolates using the macro broth dilution method against six commonly used disinfectants (Chemical disinfectants from the Guideline for Disinfection and Sterilization in Healthcare Facilities, 2008). Benzalkonium chloride (BAC), sodium hypochlorite, glutaraldehyde, CHX, (all from Sigma-Aldrich Chemicals Pvt. Ltd, India) hydrogen peroxide and ethyl alcohol (both from Thermo Fisher Scientific India Pvt. Ltd.). The appropriate concentration of the disinfectants was prepared by adding distilled water to give the strength of the chemicals as per the effective strength of disinfectant (Rani et al., 2017). Serial twofold dilutions of the disinfectants (4% CHX, 2% Glutaraldehyde, 7.5% hydrogen peroxide, 1% sodium hypochlorite and 0.1% BAC, and 70%–30% ethyl alcohol) were prepared and mixed with the inoculum which was prepared by mixing 1–2 colonies in normal saline to match 0.5 McFarland standard (1.5 × 10^8^ c.f.u. mL^−1^). Bacterial suspensions at the log growth phase were incubated with serial dilutions of disinfectants in tubes and incubated at 37°C overnight to observe visible growth. For quality control, *Escherichia coli* ATCC^®^ 25922 and *Klebsiella pneumoniae* ATCC^®^ 70063 were used.

### 2.5 Determination of MBC

The MBC analysis was performed using a modified version of the method described in the BSAC Susceptibility Guide Testing (Andrews, 2001). Following the MIC incubation period, MBC was determined by plating an aliquot of 10 μL on nutrient agar plates from each tube demonstrating no visible growth. Resultant colonies were counted after incubation at 37°C overnight. The MBC endpoint was defined as the lowest concentration of the antimicrobial agent showing at least a 99.9% killing of the initial inoculums where no visible growth of the bacteria was observed.

### 2.6 Antibiotic susceptibility testing profile

Susceptibility toward amoxicillin/clavulanic acid (AMC, 20/10 μg), gentamicin (GEN, 10 μg), ciprofloxacin (CIP, 5 μg), levofloxacin (LE, 5 μg), ceftazidime (CAZ, 30 μg), cefepime (CPM, 30 μg) ceftriaxone (CTR, 30 μg), cotrimoxazole (TMP-SMX 1.25/23.75 μg), piperacillin/tazobactam (PTZ, 100/10 μg), imipenem (IPM, 10 μg), meropenem (MEM, 10 μg), amikacin (AK, 30 μg), ertapenem (ETP 30 μg) (HiMedia Laboratories Pvt. Ltd, India) was tested by Kirby Bauer disc diffusion method. For quality control, *E. coli* ATCC^®^ 25922 and *K. pneumoniae* ATCC^®^ 70063 were used. Results were interpreted according to CLSI guidelines 2024 (Clinical Laboratory and Standards Institute, 2024).

### 2.7 Change in MIC under selective pressure of disinfectants

Determination of MIC under selective pressure of disinfectants was done to note changes in MIC if any. For this, organisms were grown in the presence of the disinfectants. The specific MIC chosen were, half of the MIC value for subinhibitory concentration and twice the MIC for supra-inhibitory concentration. Alcohol (ethyl), Hypochlorite, CHX, and BAC were used for this experiment. MIC was performed in cation-adjusted Mueller Hinton broth (CAMHB) containing tubes with specific concentrations of disinfectants and incubated at 37°C as detailed before. MIC determination was repeated after every 7 days of exposure for a period of 30 days (Merchel Piovesan Pereira et al., 2021).

### 2.8 Evaluation of formation of the biofilm in the presence and absence of disinfectants

Biofilm forming capacity of the isolates were tested with and without CHX and BAC at different concentrations. Briefly, overnight cultures were diluted to get 1.5 × 10^8^ colony-forming units (CFUs)/mL bacterial suspensions. In a microtiter plate containing 96-well (Tarsons Products Limited, India), the CHX-based disinfectant with concentration ranging from 4% (W/V) to 0.12% (W/V) and BAC ranging from 0.1% (w/v) to 0.02% (w/v) was diluted in freshly prepared Brain Heart infusion broth media supplemented with 0.25% glucose. Ten microliter bacterial suspension was added to each well keeping the final volume at 200 μL. As positive control the disinfectant-free medium containing bacterial suspension was used and wells with only broth medium were considered as negative controls. The activity of the CHX and BAC on biofilm formation was examined on standardized low, moderate, and high biofilm-producing strains of *Staphylococcus aureus*. After a 48h of incubation period, wells were washed thrice with sterile phosphate buffer saline (PBS) and allowed to air dry before stained with 200 μL of 0.1% crystal violet followed by 20 min of incubation at room temperature and washed twice with PBS. The excess stain was solubilized with 95% ethanol. The plates were again incubated for 20 min at room temperature (Stepanovic et al., 2007). The optical density at 578 nm (OD578) of each well was measured using a microtiter plate reader. (LisaScan^®^ EM Elisa plate reader, Transasia Bio-Medicals Ltd, India). The formation of biofilm was calculated by the following formula (Singh et al., 2017):

ODcut = ODavg of negative control + 3 × standard deviation of ODs of negative control.

OD ≤ ODcut = Non−biofilm−former

ODcut < OD ≤ 2 × ODcut = Weak biofilm former

2 × ODcut < OD ≤ 4 × ODcut = Moderate biofilm former

OD >4 × ODcut = Strong biofilm former.

Percentage reduction in biofilm formation was calculated by (Nunes S de et al., 2021):


$$\frac{O.D\;of\;control\;well-O.D.\;of\;test\;well}{O.D\;of\;control\;well}\times \;100$$

### 2.9 Whole genome sequencing

WGS was performed using Illumina platform (outsourced to Bionivid Technology Private Limited, Bengaluru, India). Fastp 0.23.0 was used for quality control, adapter trimming, and data filtering (Chen et al., 2018). alignment quality was checked by using samtools 1.20 (Kim et al., 2019). Annotation of genomes were done using SnpEff (Cingolani et al., 2012). Variant detection was done using VarScan 2.4.6 (Koboldt et al., 2009).

#### 2.9.1 WGS analysis tools

RGI-CARD (Comprehensive Antibiotic Resistance Database) and ResFinder 4.1 (https://cge.food.dtu.dk/services/ResFinder/) were used to predict resistance genes in assembled bacterial genomes. Multi-locus sequence typing (MLST) patterns were identified using MLST 2.0.9 (https://cge.food.dtu.dk/services/MLST/).

### 2.10 Statistical analysis

Statistical analysis was performed by ANOVA to compare the biofilm reduction rates, using Microsoft Excel (Version: Microsoft Corporation 2019). Results with a *p*-value ≤ 0.05 were considered statistically significant.

## 3 Results

### 3.1 Isolate details

During the microbiological surveillance, disinfectant samples from 18 separate dispensers distributed in different locations of the hospital were collected. Among these, the four bacterial strains isolated from four sites, namely, Kp1, Kp2, Pa1, and Pa2 were identified as 2 K*. pneumoniae* and 2 P*. aeruginosa.* During the same surveillance, gram-negative bacteria were not found on any of the other hospital surfaces.

### 3.2 In-use testing

The four disinfectants showed the presence of the same bacteria thus implying contamination with colony count >5. However, no contamination was detected in the unused disinfectants of the same batch.

### 3.3 Determination of MIC and MBC against selected disinfectants

The susceptibility testing demonstrated the MIC values of the CHX ranging from 1% (w/v) to 4% (w/v), for Hydrogen peroxide 0.23% (w/v) to 0.40% (w/v), for sodium hypochlorite 0.25% (w/v) to 1% (w/v), for BAC 0.10% (w/v), for glutaraldehyde 0.12% (w/v) to 0.50% (w/v), and for ethyl alcohol it was 30% against the different test organisms, respectively. The highest MBC/MIC ratio of the CHX, sodium hypochlorite, and BAC against test isolates was 2; for hydrogen peroxide and ethyl alcohol, it was 1; and for glutaraldehyde was 4. The complete data on MIC and MBC values are shown in **Table 1**.

**Table 1:** MIC and MBC values of disinfectants in studied isolates.

Sl. No	Bacterial isolates	Disinfectants											
		Chlorhexidine(4% w/v)		Hydrogen peroxide (7.5%w/v)		Glutaraldehyde (2% w/v)		Sodium Hypochlorite (1 % w/v)		BAC (0.1% w/v)		Ethyl Alcohol (70% v/v)	
		MIC	MBC	MIC	MBC	MIC	MBC	MIC	MBC	MIC	MBC	MIC	MBC
1.	Kp1	1.00	2.00	0.46	0.46	0.12	0.25	0.25	0.50	0.10	0.10	30	30
2.	Kp2	1.00	2.00	0.46	0.46	0.50	1.00	0.25	0.50	0.10	0.10	30	30
3.	Pa1	2.00	4.00	0.46	0.46	0.25	1.00	0.50	0.50	0.10	0.10	30	30
4.	Pa2	4.00	8.00	0.46	0.46	0.50	1.00	1	1	0.10	0.10	30	30
5.	*P.aeruginosa* (ATCC27853)	0.50	1.00	0.23	0.23	0.25	0.5	0.25	0.50	0.10	0.10	20	20
6.	*K.pneumoniae* (ATCC70063)	0.50	1.00	0.23	0.23	0.25	1	0.50	1	0.20	0.40	40	40

### 3.4 AST profile

Among the 2 K*. pneumoniae* isolates, resistance was noted for AK, LE, IPM, MEM, and TMP-SMX against Kp2 isolate. The other isolate was susceptible to all antibiotics. For the two *P. aeruginosa* isolates, resistance was noted against PIP, PTZ, LE, MEM, IPM, and CAZ for both the isolates.

### 3.5 Change in MIC under selective pressure of disinfectants

Changes in MIC and MBC increased to twofolds for Kp2 against BAC after 21 days of exposure. However, no MIC and MBC changes were seen in the other isolates against the tested disinfectants (BAC, CHX, sodium hypochlorite, and ethyl alcohol).

### 3.6 Evaluation of biofilm formation in the presence and absence of disinfectants

**Figure 1:**
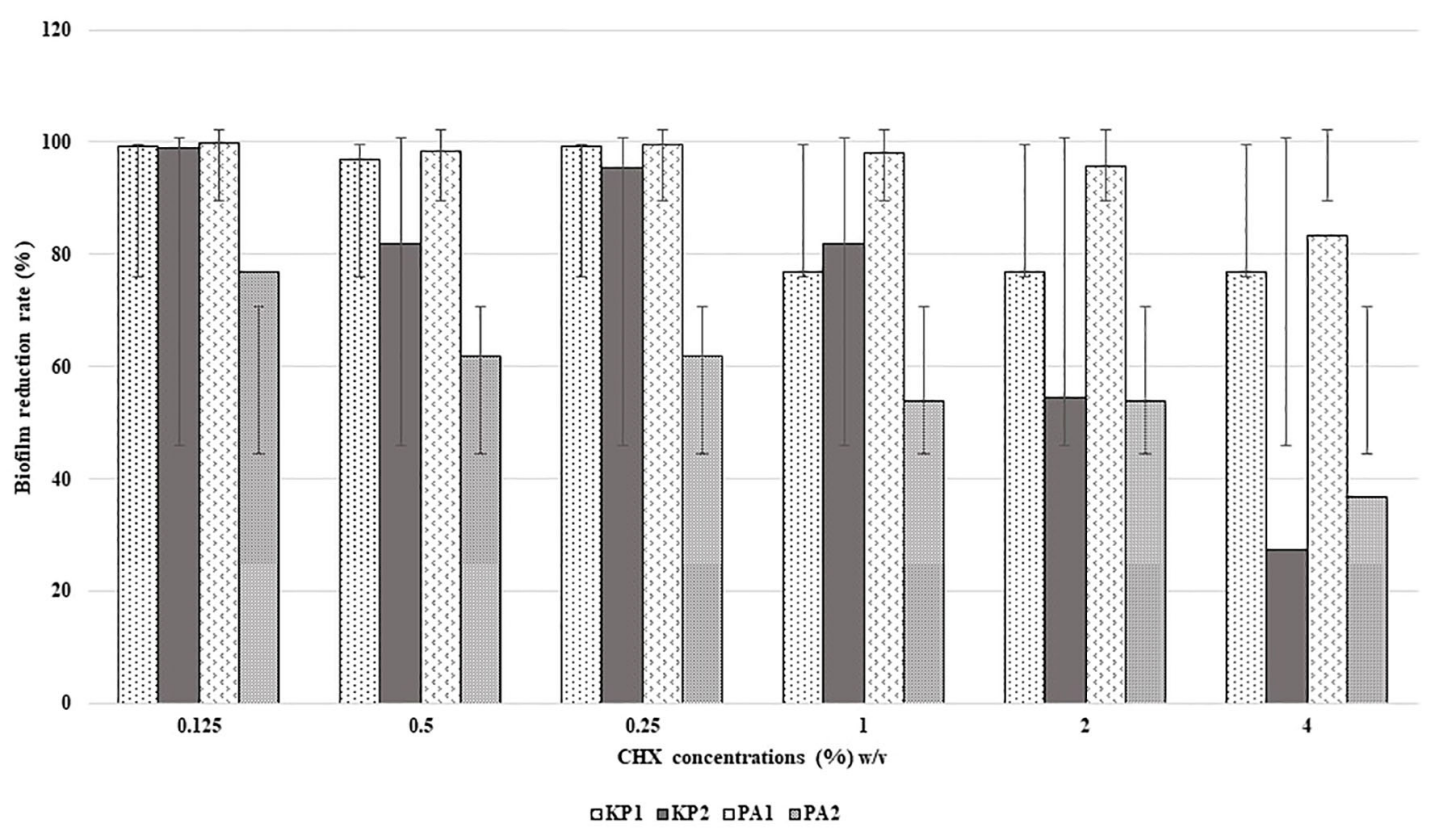
Reduction in biofilm formation according to concentration of CHX.

**Figure 2:**
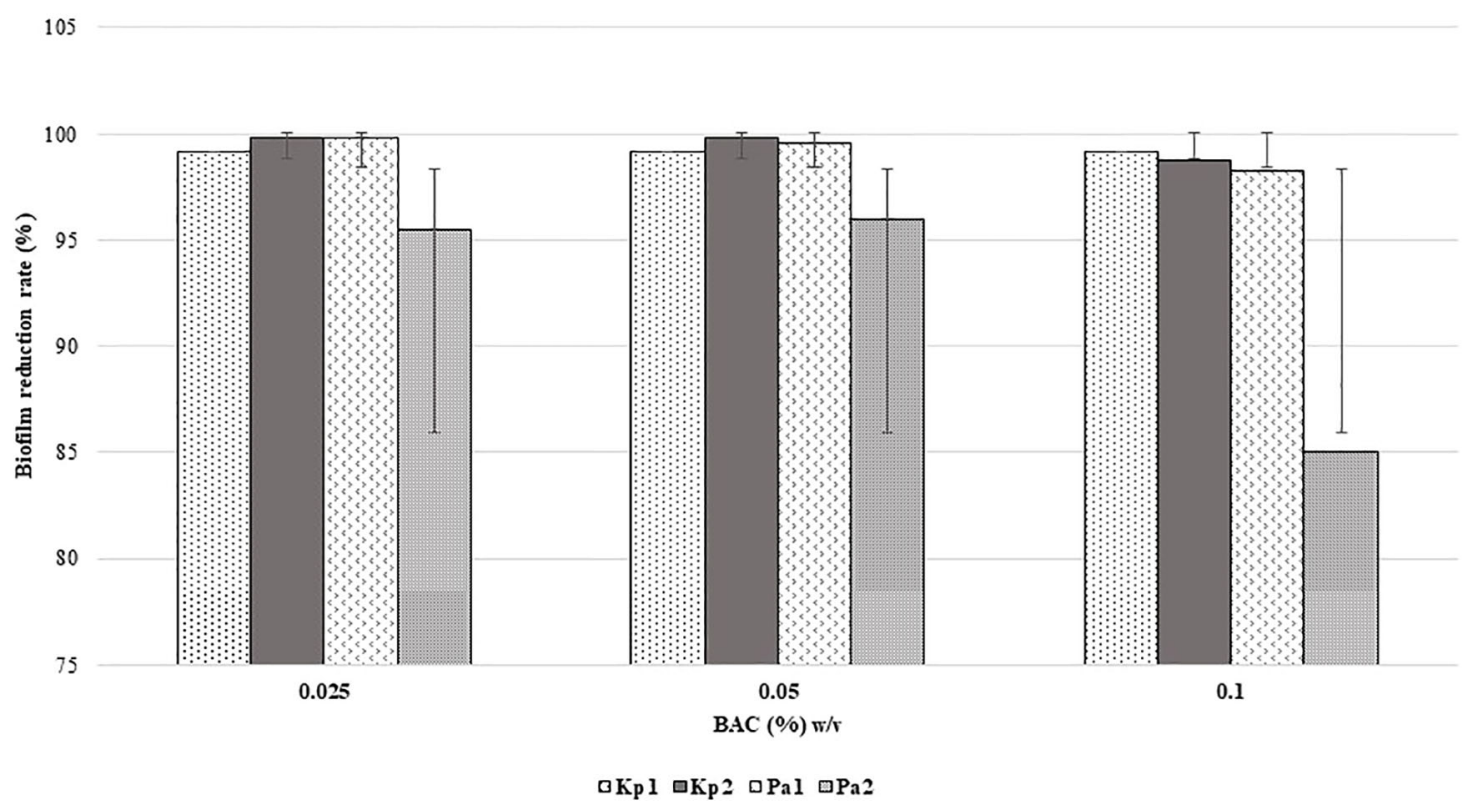
Reduction in biofilm formation according to concentration of BAC. (*p* = 0.05, two-way ANOVA applied).

All the isolates were found to be high biofilm producers compared to high and moderate biofilm-producing strains, in the presence and absence of disinfectants. The rate of reduction of biofilm formation decreased with an increase in the concentration of disinfectants as shown in **Figures 1** and **2**. BAC showed a significant formation (*P* = 0.05) of biofilm at higher concentrations.

### 3.7 Analysis of WGS results

Isolate Kp1 was found to carry *KpnE* of SMR family, *leuO*, *acrA*, and *marA* of RND family. Sequence type (ST) was unknown of this isolate. MLST for Kp2 was ST 2676 and was found to harbor QAC-resistant gene *qacE* and antibiotic-resistant genes like β-lactam resistant gene *bla*_OXA-1_, aminoglycoside resistant gene *aph*(3’)-la, *bla*_DHA-1_, *bla*_OKP-A_, fosfomycin resistant gene *fos*A, quinolone-resistant gene *qnrB1, qnrB4*, sulfonamide resistant gene *sul-1*, trimethoprim resistant gene *dfrA*, and tetracycline-resistant gene *triA*. Isolate Pa1 belonged to ST 1047 and showed presence of antibiotic-resistant genes like *aph*, *crpP*, *bla*_PAO_, *bla*_OXA-488_, *sul-1*, *fosA*, and *catB*. Pa2 had *tri* genes, *mex* genes, *OprM*, outer membrane porins, and *yajC* genes conferring resistance to RND family, *soxR* conferring resistance to SHV family, *PmpM* conferring resistance to MATE family. Antibiotic-resistant genes of β-lactam family *bla*_DIM-1_, *bla*_PAO,_ *bla*_OXA488,_ for aminoglycoside family *ant*(2’’)-Ia, *aph* (6)-*Id* were detected and ST of this isolate was ST 4249. Resistant genes of Kp1, Kp2, Pa1, and Pa2 have been shown in **Tables 2**–**5** respectively and resistance mechanism along with genes for Kp1, Kp2, Pa1, and Pa2 has been shown in representative **Supplementary Figures S1–S4** respectively.

**Table 2:** Distribution of resistant genes for Kp1.

Isolates	Antibiotic resistance ontology (ARO term)	AMR gene family	Drug class	Resistance mechanism
Kp1	*KpnF*	Small multidrug resistance (SMR)	Disinfecting agents and antiseptics	Antibiotic efflux
	*LeuO*	Major facilitator superfamily (MFS)	Disinfecting agents and antiseptics	Antibiotic efflux
	*marA*	Resistance nodulation division family (RND)	Disinfecting agents and antiseptics	Antibiotic efflux
	*LptD*	ATP binding cassette superfamily (ABC transporter)	Carbapenem	Antibiotic efflux
	*SHV-11*	SHV beta lactamase	Carbapenem	Antibiotic inactivation
	*MdtQ*	Outer membrane porin (Opr)	Cephalosporin	Reduced permeability to antibiotic

**Table 3:** Distribution of resistant genes for Kp2.

Isolates	Antimicrobial	Class	Resistant phenotype	Resistant gene
Kp2	Hydrogen peroxide	Peroxide	No resistance	No resistance
	Ethidium bromide	QAC	Resistant	*qacE* (*qacE*_X68232)
	BAC	QAC	Resistant	*qacE* (*qacE*_X68232)
	Chlorhexidine	QAC	Resistant	*qacE* (*qacE*_X68232)
	Cetylpyridinium	QAC	Resistant	*qacE* (*qacE*_X68232)

**Table 4:** Distribution of resistant genes for Pa1.

Isolates	Antimicrobial	Class	Resistant phenotype	Resistant gene
Pa1	streptomycin	aminoglycoside	Resistant	*aph(3’)llb*
	ciprofloxacin	quinolone	Resistant	*crpP*
	amoxicillin	β-lactam	Resistant	*bla*_PAO_
	ampicillin	β-lactam	Resistant	*bla*_PAO_
	cefepime	β-lactam	Resistant	*bla*_PAO_
	ceftazidime	β-lactam	Resistant	*bla*_PAO_
	unknownβlactam	β-lactam	Resistant	*bla*_OXA488_
	sulfamethoxazole	Folate pathway antagonist	Resistant	*Sul1*
	fosfomycin	fosfomycin	Resistant	*fosA*
	chloramphenicol	amphenicol	Resistant	*catB7*

**Table 5:** Distribution of resistant genes for Pa2.

Isolates	Antibiotic Resistance Ontology (ARO Term)	AMR gene family	Drug Class	Resistance mechanism
Pa2	*triA, triB, triC*	RND	Disinfecting agents and antiseptics	Antibiotic efflux
	*OprM*	RND	Disinfecting agents and antiseptics	Antibiotic efflux
	*PmpM*	Multidrug and toxic compound extrusion (MATE)	Disinfecting agents and antiseptics	Antibiotic efflux
	*ParS, ParR*	RND	Disinfecting agents and antiseptics	Antibiotic efflux, reduced permeability to antibiotic
	*SoxR*	Sulfhdryl variable β-lactamase (SHV)	Disinfecting agents and antiseptics	Antibiotic target alteration, Antibiotic efflux
	*MexY, MexQ, MexP, MexK, MexJ, MexL, MexG, MexH, MexI, MexV, MexW, MexZ*	RND	Disinfecting agents and antiseptics	Reduced permeability to antibiotic
	*OpmE, OpmD, OpmH*	RND	Disinfecting agents and antiseptics	Antibiotic efflux
	*YajC*	RND	Disinfecting agents and antiseptics	Antibiotic efflux
	*bla*_DIM-1_	amoxicillin	β-lactam	Reduced permeability to antibiotic

## 4 Discussion

This study clearly demonstrated the ability of the bacterial contaminants to persist in the presence of the disinfectants, particularly CHX and BAC. Bacterial tolerance to BAC increased on prolonged exposure to the disinfectant and the rate of reduction in bacterial biofilm formation decreased with increasing concentration of disinfectants. On these aspects, the study is important because there is very limited data on bacterial responses towards disinfectants. In this context, the present study highlighted the following points.

Firstly, there is lack of standard definition/criteria of disinfectant resistance in the scientific community, which could be one of the reasons for scarce literature on this aspect (van Dijk and Verbrugh, 2022). Studies have either compared MIC/MBC values of the test isolates with those of the standard isolates or have studied their exact MIC values at varying disinfectant concentrations. In this study, though MIC was not overtly raised in any of the isolates MBC was double the MIC for CHX, glutaraldehyde, and sodium hypochlorite (isolates Kp1, Kp2). This increase in MBC could hint towards the increasing “tolerance” to disinfectants in these isolates (Nordholt et al., 2021). The MIC/MBC values of all the isolates against CHX, hydrogen peroxide, and alcohol were more as compared to the standard strains. There have been reports of emerging disinfectant resistance across the globe (Tong et al., 2021). Varying resistance rates (1–256µg/mL) for *P. aeruginosa* against CHX has been reported (Mal et al., 2016). Similarly, a study on 126 isolates of *K. pneumoniae* showed high MIC values and decreased susceptibility in 90% of isolates (Naparstek et al., 2012). A recent Indian study documented higher MIC values for 12.5% and 50% of *K. pneumoniae*, 80% and 20% of *P. aeruginosa* against 70% ethanol and sodium hypochlorite respectively (Gupta and Bhatia, 2018). It has been seen that the concentrations at which disinfectants are used in the hospital are considerably higher than their MIC values. Yet, they do not assure successful disinfection procedure (van Dijk and Verbrugh, 2022).

For BAC, comparable MIC/MBC values were noted against the standard isolates. Both lower (0.02–0.2) and higher MIC (30–120) values have been noted in studies and higher MIC (30–120) has been noted in studies in India and the USA (Rajamohan et al., 2010; Gupta and Bhatia, 2018). However, it was interesting to note that one *K. pneumoniae* (Kp2) isolate showed double increase in the MIC and MBC of BAC following 21 days of exposure to supra-inhibitory concentration of BAC. It has been advocated that exposure to subinhibitory concentration of disinfectants, which is mostly seen at the periphery of the disinfected area, can initiate several stress responses in the bacteria (Koboldt et al., 2009). On WGS, this isolate Kp2 had shown the presence of *qac* genes the expression of which could have accounted for this variation in MIC/MBC. In this context, study had shown 53% presence of *qacE* gene responsible for biocide resistance in *K. pneumoniae* isolates (Abuzaid et al., 2012). The *qac* genes have been reported widely for causing disinfection tolerance. This plasmid-mediated group of genes are also responsible for dissemination of disinfection resistance. The *qacE* gene has been located often in association with integron, thus increasing the risk of horizontal transmission (Tong et al., 2021).

Thirdly, differences in disinfectant susceptibility might be accompanied by antibiotic resistance by a phenomenon of cross-resistance. As shown by AST and WGS, these isolates harbored various AMR genes of importance. In this study, three of the four isolates were resistant to multiple antibiotics. WGS revealed several genes that could account for this resistance. The presence of multidrug-resistant (MDR) genes in clinical isolates of *K. pneumoniae* and *P. aeruginosa* had been a known fact. One of the isolate (Kp1) carried multiple genes for antibiotic efflux pump like *LptD*, *SHV-11*, *MdtQ*, which could explain the resistance to beta lactam agents like cephalosporins and carbapenems in this isolate. The *Pseudomonas* isolates were widely resistant to beta lactam antibiotics, aminoglycosides, fosfomycin and also possessed genes like *bla*_PAO_, aminoglycoside phosphotransferases corresponding to their phenotypic resistance. In this context, the study also revealed the presence of *bla*_DIM-1_ gene in Pa2, a less studied carbapenem-encoding gene. In India, *bla*_DIM-1_ was first identified in a *P. stutzeri* strain collected in 2000 and reported in 2014 (Deshpande et al., 2014). As carbapenems are considered to be one of the last resorts of antibiotics for *P. aeruginosa* infections, the identified *bla*_DIM-1_ gene in this study is quite concerning.

Besides, several disinfectant resistance genes conferring resistant to the efflux pump families were also found in these isolates. They carried genes from all the five efflux pump membrane protein families (RND, MFS, MATE, SMR, and ABC). These efflux pumps have been noted to affect disinfection activity of the biocides against the organisms harboring them (Tong et al., 2021). As efflux pumps are one of the most effective resistance mechanisms that bacteria employ against stress, it could be possible that the diversity in these pumps was an adaptation in these isolates to survive within the disinfectant environment. Additionally, Pa1 belonged to ST 1047, which is a globally circulating clone of MDR strains especially in the Southeast Asian region (Tada et al., 2019; Takahashi et al., 2021).

Last, the study reinstated the importance of biofilm formation as an adaptation strategy for survival in the hospital environment. All the four bacterial isolates were high biofilm producers both in the presence and absence of disinfectants namely CHX and BAC. When biofilm production was assessed at increasing concentration of these disinfectants, it was very interesting to note that the rate of reduction of biofilm formation decreased as the concentration of disinfectants increased. This phenotypic presentation could be one of the most important strategy of these bacterial isolates to survive in the disinfectant environment. It could be possible that the possession of several types of efflux pumps as detected by WGS, which are implicated in biofilm production, could have been activated at subinhibitory concentration of disinfectants thus promoting more biofilm formation (Sekiya et al., 2003). A study on expression profile of these pumps under these circumstances, which was not within the scope of the present study, could confirm the prediction. This finding also raises concern as use of disinfection in appropriate dilutions is an existing issue. If bacteria like *P. aeruginosa, K. pneumoniae* can survive the working concentrations of disinfectants through biofilm formation, rapid emergence of resistance is inevitable. However, unless there is considerable baseline data on disinfectant resistance/tolerance that is widely prevalent, it would be very difficult to comment on whether disinfectant resistance is emerging or already existing.

The study was not without limitations. While we could not locate the exact area where the bacteria were residing in the disinfectant container which could have translated the expected finding into the real scenario. We could only access the biofilm formation ability of the organisms, while visualization of the real biofilm structure would have added more value. Moreover, experiments of efflux pumps could not be done which could have confirmed the phenotypic presentations. Nevertheless, the study is of utmost importance because to the best of the knowledge, it is the first study on adaptation of bacterial isolates in the disinfectant environment of the hospital, highlighting the different strategies adopted by the bacteria to persist.

## 5 Conclusion

Bacterial isolates, 2 K*. pneumoniae*, and 2 P*. aeruginosa* were found to survive in CHX-based disinfectants in the hospital environment. All the four isolates had increased MIC/MBC against glutaraldehyde and sodium hypochlorite. Exposure to supra-inhibitory concentration of BAC resulted in doubling of MIC/MBC. All the isolates were strong biofilm producers both in the presence and absence of disinfectants with reduced rate of reduction in biofilm formation at increasing concentrations. WGS revealed multiple AMR genes including *bla*_DIM-1_, disinfectant-resistant gene and efflux pump genes in these isolates. The study emphasized the various adaptation strategies of these isolates for survival in disinfectant environment, thus posing a huge challenge for their control in the hospital environment.

## Data Availability

The raw data supporting the conclusions of this article will be made available by the authors, without undue reservation.
